# Do Managerial Ties Help or Hinder Corporate Green Innovation? The Moderating Roles of Contextual Factors

**DOI:** 10.3390/ijerph19074019

**Published:** 2022-03-28

**Authors:** Yu Zhang, Yajuan Wang

**Affiliations:** 1 Business School, Hohai University, Nanjing 211100, China; yuyuzhang88@sina.com; 2 International Business School, Shaanxi Normal University, Xi’an 710119, China

**Keywords:** green innovation, managerial ties, environmental regulations, competitive intensity

## Abstract

Green innovation has significant implications for firms’ financial, environmental, and social performance. However, its externalities may inhibit the proactive involvement of firms in such initiatives. In this study, we examined the roles of two types of managerial ties (i.e., business and political) in green innovation and further investigated the moderating effects of two types of contextual factors (i.e., environmental regulations and competitive intensity). By conducting an empirical study using survey data from 218 samples, we confirm that business ties positively affect green innovation while political ties have an inverted U-shaped effect. Moreover, the relationship between managerial ties and green innovation is contingent on specific context settings. Our results show that the environmental regulations enforced by the government strengthen both the effects of business and political ties, while the competitive intensity has no effect on the relationship between business ties and green innovation; however, it sharpens the curvilinear effect of political ties.

## 1. Introduction

Currently, in response to the problems of resource shortage and environmental degradation, as well as the increased public awareness of environmental protection, corporate green innovation has aroused the common interests of practitioners, scholars, and policymakers [1], specifically regarding more environmentally friendly initiatives such as energy saving, pollution prevention, waste recycling, and eco-design [2,3,4]. Traditional practices, products, and processes have been updated through green innovation to be more resource-efficient and less detrimental to the natural environment [2,5,6]. Apart from these ecological benefits, green innovation has been shown to provide enterprises with distinctive advantages through improved production efficiency [7,8,9,10], product favorability [11], and corporate image [1,12]. Therefore, green innovation is considered a critical means by which to achieve high-quality and sustainable development [2,3] and improve public health and human well-being [13,14].

Given the growing importance of green innovation, researchers have endeavored to identify its driving forces from various perspectives [15,16]. Some of them consider “turning green” efforts as an outcome of macro-factors external to the firm, including environmental regulations [4,17], stakeholder pressures [1], and technological factors [13,15]. Another stream of the literature has examined the intra-firm mechanisms that underlie the adoption of green innovation at the micro-level, such as the firm’s environmental ethics [18], organizational capability [19,20], and environmental management systems [21]. Despite these insightful studies, we noticed that research on the influence of meso-level determinants, such as the social networks the firms are embedded in, has not been commensurate with its importance in current green innovation literature [2].

Green innovation represents a modification in a firm’s business model and operating routines, and thus certain resources and competences, usually beyond what a firm possesses now, are required to adapt to the challenges that ensue [21,22]. This fact highlights the pertinence of interactions between a firm and its external players in the firm’s journey toward green development [2,6,23,24,25,26]. According to social network theory, the managers’ ties built with various social actors could be utilized to acquire the necessary resources and achieve the desired goals. Previous studies have identified different types of managerial ties, including those with business organizations and with political entities, and recognized their value in improving a firm’s standard innovations [27] and performance [28,29]. Yet, how they would affect the pro-environmental innovation has not been explicitly discussed.

Furthermore, the development of social network theory and the integration of contingency view has warned that the effectiveness of these ties may not be uniform across all contexts [28]. In this vein, we presume that the potential impacts of managerial ties on green innovation would be contingent on the extent of environmental regulations that have been enforced by the government and the competitive intensity of the market. On the one hand, regulation constitutes an important part of the institutional context wherein firms operate. It affects a firm’s prosocial considerations from a legitimacy-based perspective by constraining what actions are acceptable and supportable [30,31]. On the other hand, competitive intensity is a critical characteristic of task context where firms strive to exemplify economic fitness [32]. It is also likely to have an impact on the firms’ environment-related choices from an efficiency-based perspective by defining what actions are profitable [33]. Therefore, how these two types of contextual factors affect the role of managerial ties in green practices deserves further investigation to deepen our knowledge in this field.

To fill these research gaps, we draw on social network theory, contingency view, and green innovation literature to develop an integrated model to (1) investigate the effects of two types of managerial ties, including business and political ties on green innovation initiatives, and (2) examine the moderating effects of environmental regulations and the competitive intensity on the relationships between managerial ties and green innovation. By conducting an empirical study using survey data comprising 218 sample manufacturers from China, we find that business ties are positively related to green innovation, whereas political ties have an inverted U-shaped effect. In addition, environmental regulations positively moderate the effect of business ties while sharpening the inverted U-shaped effect of political ties. On the other hand, competitive intensity has no effect on the relationship between business ties and green innovation, yet it magnifies both the positive and negative effects of political ties.

The remainder of this paper is structured as follows: Section 2 reviews the relevant literature. Section 3 proposes specific hypotheses. Section 4 describes methods and results of the empirical analyses. Section 5 discusses our findings and outlines their potential theoretical contributions. Section 6 briefly highlights the essential conclusions, summarizes their implications for managers and policymakers, and identifies limitations and avenues for future research.

## 2. Literature Review

### 2.1. Green Innovation

Green innovation is an important avenue for reaching ecological-specified sustainable goals in a cost-effective way [34,35,36]. It involves modifying existing elements or exploring new environmentally friendly elements for various aspects such as product and process for the purpose of reducing environmental damages and natural resource consumption in a firm’s operations [11,35]. In addition, corresponding organizational and management innovations may also be included [8].

It has been increasingly recognized that firms should consider customer and business values while decreasing negative environmental impacts [9,10]. Green innovation has enabled the firms to achieve a balance between economic development and ecological improvements goals [6,24,36,37]. For example, developing green products that use pro-environmental materials and packaging and that are easy to recover and reuse not only reduce waste and the consumption of raw materials [16,26], but also cater to customers who care about healthier goods and protecting the environment [9]. Similarly, implementing green processes that make better use of energy, recycle resources, and mitigate toxicity can help a firm improve their efficient use of resources [10], reduce production costs [9,11], and alleviate pollutant emissions [2,3,38]. A large body of literature has shown that green innovation has been a source of competitive advantage and sustainable development for enterprises [3,22].

However, there are debates over the benefit of green innovation. Its public good character causes the peculiar “double externality” problem and has constrained firms from fully appropriating the profits [35]. On the one hand, the knowledge spillovers of innovation in general are also common in green innovation. On the other hand, as compared to traditional innovations, green innovation leads to environmental spillovers. While reducing environmental costs for the society, innovations with eco-benefits have led to unexpectedly higher costs and risks that have to be borne by a single firm. First, green innovation integrates solving different techno-economic and environmental issues via the specific innovation [24,35,37]. Such a complex project entails substantial resource dedication and incurs significant opportunity costs [7]. Second, the transition toward green development has typically involved a long-term exploratory process and investment period without the guarantee of (quick) success [39,40]. Finally, as suggested by the traditional view of corporate environmentalism, environmental management could cause inefficiencies and productivity losses by imposing constraints on industry behaviors [22,41]. Furthermore, plenty of consumers are unwilling to pay more for green product attributes [22]. Accordingly, it has been found that both externalities are likely to result in a sub-optimal investment in environmental initiatives [8,24,42].

Given the bright and dark sides of green innovation, it would be appealing to delve into the question of how to overcome the potential challenges and promote a firms’ willingness and capacity to embrace green innovation.

### 2.2. Managerial Ties

The market failures brought on by the externalities of green innovation call on firms to pay more attention to the role of nonmarket forces. In such situations, their managers’ social ties have emerged as an important mechanism that could be leveraged to the benefit of green innovation and raise the incentives for firms to perform such initiatives [29]. According to social network theory, firms are closely embedded in networks of ties, and their strategic decisions and business activities are inevitably influenced by those connections [43]. Managerial ties, namely, the executives’ boundary-spanning activities and associated interactions with external entities, have proven to be critical for firms’ resource acquisition, innovative activities, and performance [28].

There are two different types of managerial ties: business ties and political ties; the former refers to interactions and social relationships developed with business players in the market such as suppliers, buyers, competitors, and collaborators, while the latter are informal connections built with leaders and officials of government institutions and regulatory organizations [29,44]. These two types of ties have diverse resource-bridging and adaptive capabilities, trigger disparate willingness to sustain the relationships, and force firms to evaluate and respond to situations differently [18,45,46]. Therefore, we argue that they both are critical to understand the variations in firms’ green innovation initiatives, yet in heterogeneous ways.

**Figure 1:**
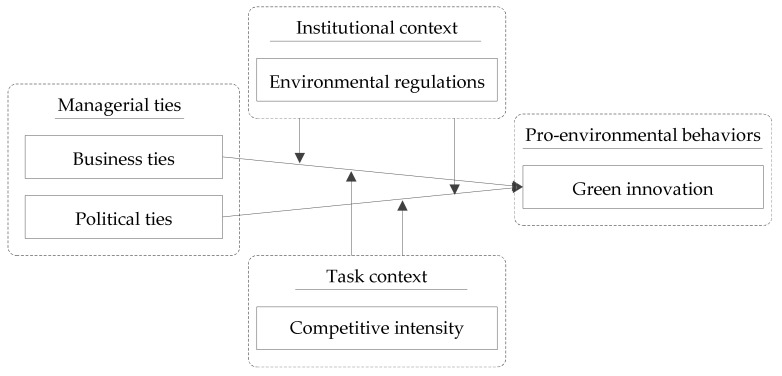
Conceptual framework.

Based on the review of previous relevant literature, we present the conceptual framework of this study in Figure 1.

## 3. Hypotheses

### 3.1. Effects of Managerial Ties on Green Innovation

#### 3.1.1. Effect of Business Ties

We anticipate that business ties have the potential to facilitate green innovation. First, managers’ relationships with various business players in the market could help firms identify, acquire, and deploy external resources required for green innovation [47]. For example, the intimate relationships with upstream partners could provide knowledge concerning more environmentally friendly materials, components, and logistics [34]. Close ties with the buyers could garner their support when introducing green products to the market and yield valuable insights into the customers’ green needs. Healthy ties with both competitors and collaborating partners could inform a firm of cutting-edge green technologies, green trends in the industry, and environmental ethics [18,48].

Second, the inter-firm relationships create opportunities for cooperative green innovation and can reduce transaction costs in the process. Close and frequent interactions function as an informal enforcement mechanism to coordinate the collaboration, since the relational norms, trust, and reciprocity developed between firms [48] could lower the risk of opportunism and facilitate collective efforts to achieve shared environmental goals [2,5].

Third, the close business ties improve a firms’ ability to manage risks and adapt to changes [2,23]. These ties increase the willingness to share risk among connected firms, foster the executives’ risk-tolerance, and thus could encourage more adventurous behaviors such as active involvement in green innovation [49].

Fourth, social networks with various business organizations assist the firms in obtaining commercial legitimacy and credential of social status in the business community [50], which are instrumental for alleviating the resistance and paving the way for transforming existing processes or products. Therefore, we propose:

**Hypothesis 1** **(H1).** *Business ties have a positive relationship with green innovation*.

#### 3.1.2. Effect of Political Ties

Governmental institutions have been another important source of external resources and opportunities for the firm [51]. Increases in political ties have initially created a favorable condition for corporate green innovation. First, political ties have offered shortcuts to government financial support and favorable treatment [52,53], alleviating the costs and financial constraints brought about by green innovation [52]. Political capital accessed through close ties with regulators has also included information about the latest and even future trends of environmental policy, industrial development programs, and government grants related to environment protections, giving the firm a privilege and direction in which to exploit the opportunities of green innovation [34,51]. In addition, close relationships with governmental contacts have generated social credentials that could stimulate generalized trust in and recognition of a firm among external stakeholders, attracting other resources such as financing opportunities and intellectual capital for green innovation [54]. Second, political connections have also served as a kind of environmental legitimacy constraint. Firms with governmental ties have attracted greater external attention and face higher expectations [54], pressuring them to take on more social responsibilities and respond to growing environmental concerns [55]. In addition, those firms might have more duties to assist the government in meeting the environmental indicators of performance assessment [54]. Third, political ties have enabled firms to share the risk of green innovation with the government, which has served as an informal insurance and motivated more risk-taking in green innovation [56].

However, as political ties increase beyond a certain point, the positive effect begins to decline. First, over-investment in political relationships in such forms as gift-giving, banquets, and philanthropical activities may divert resources away from environmental concerns [52] and crowd out green input [57]. Second, heavy reliance on privileges and shields from the market pressure provided by the government may hamper the firm’s motivation and capability to compete through its endogenous strengths over time. Such firms are more likely to rest on their laurels and remain in extant trajectories, rather than pursue continuous improvements and innovations, let alone environmental innovation with higher levels of uncertainty [45,58]. Third, political ties tend to be more transient [29]. Hence, the firms excessively seeking to develop relationships with the government might be more inclined to exploit their political resources for immediate benefits rather than engage in environment-related initiatives that provide few returns in the short-term [52]. In addition, it might be more convenient for such firms to escape the environment-related pressure from the public by means of muting or controlling media and journalists through administrative approaches [34]. Therefore, we propose:

**Hypothesis 2** **(H2).** *Political ties have an inverted U-shaped relationship with green innovation*.

### 3.2. Moderating Effects of Contextual Factors

A firm’s behaviors and functioning are subject to the demands and the requirements originating from both the institutional and task contexts under which it has operated [59]. As specific characteristics of these two types of forces, we identify environmental regulations and competitive intensity, respectively, in this study to be the possible contingent factors that might influence the effects of managerial ties on green innovation.

#### 3.2.1. Moderating Effect of Environmental Regulations on Business Ties

Environmental regulations refer to a series of mandatory requirements from the administrative powers for firms to improve their environmental performance, and also specify sanctions for environmental violations [22,60,61,62]. They include both policy with-holders (i.e., sticks) such as environmental taxes, charges, and fees that use tight measures to set the boundaries on environmental damage, and policy enhancers (i.e., carrots) such as government grants, subsidies, and other financial incentives for environmental innovations [6].

Environmental regulations compel firms to be attentive to environmental issues while increasing their perceived value of green investments [7]. To obtain regulatory legitimacy and avoid penalties for poor compliance with regulations, firms should consider more non-economic responsibilities in their operations [11]. They currently have greater incentives to resort to their business ties for resources such as clean technologies and cooperative opportunities for the sake of green innovation. Environmental regulations have also provided informative and guideline values [63]. Detailed policies have provided clear standards for the firms to discover how to carry out their green innovations, such as which pollution control technologies to use and what specific targets to achieve [6,60,61]. Therefore, with more rigorous environmental regulations, firms have been more receptive to new green ideas and more likely to take advantage of business ties for green innovation [62].

Environmental regulations imposed by the government have also raised the related consciousness of the public. Moreover, policy enhancers such as subsidies and special funds for green R&D, the awards related to environmental protection, and the monetary incentive for consumers to use products with sustainable features, improve green development and eco-product pursuits in society [1,63], consequently encouraging network partners to explore green innovation and share the risks together. In addition, stringent regulations form explicit criteria for evaluating a firm when conducting business. Information about a firm’s (non)compliance with rules has spread more quickly among firms connected by strong ties and thus significantly influenced its network reputation [29]. Accordingly, firms with greater business ties would be more apt to conduct pro-environmental activities such as green innovation to abide by regulations. Therefore, we propose:

**Hypothesis 3a** **(H3a).** *The positive relationship between business ties and green innovation would be stronger when environmental regulations are more stringent*.

#### 3.2.2. Moderating Effect of Environmental Regulations on Political Ties

Environmental regulations might have an impact on the relationship between political ties and green innovation as well. First, higher regulatory pressure strengthens the political acumen of firms relying on political ties. The aggressive promulgation of environmental laws and regulations signals that the government is increasingly keen to protect the environment [22,64]. Resource dependence on the government has propelled firms to be more attentive to these concerns and more active in their allocation of resources for environmental needs [51,65]. Adopting green innovation to respond to the environmental regulations has also been beneficial in maintaining relationships with government authorities. Second, under stringent regulations, firms with political ties enjoy more advantages in undertaking proactive environmental strategies. They obtain more information from the regulations and are better able to interpret them so as to guide their environmental innovations and avoid detours [29]. It has also been easier for those firms to acquire the regulatory incentives provided by the government for stimulating green innovations [58,66].

However, environmental regulations may also augment the negative effect of extensive political ties on green innovation. First, firms relying heavily on political ties may lack the enthusiasm or ability to innovate and could be eager to turn their political ties into immediate benefits. Coercive stipulations and corresponding penalties have intensified their resistance towards environmental issues [60]. Then, the political ties have been more likely to be exploited as a sort of protective umbrella, under which firms attempt to circumvent certain environmental rules and regulations [12,51]; for example, they could alleviate government inspections and penalties, and take advantage of the loopholes in regulations. Such firms are more inclined to pursue passive environmental strategies, as compared to green innovation, and engage in perfunctory or even deceptive green initiatives, presenting symbolic compliance to the regulations [7]. Second, when there are more policy enablers for corporate environmental commitment, although firms having extensive political ties can obtain financial support and incentives from the government with greater ease, they are more likely to use such resources for other more profitable purposes without detection or penalties [52]. Third, strong political ties provide firms with opportunities to slow the pace of environmental legislation, or even influence the enactment of regulations, especially local government policies, in their favor, yet against their competitors [18]. These initiatives would ultimately reduce firms’ investments in green innovation. Therefore, we propose:

**Hypothesis 3b** **(H3b).** *The inverted U-shaped relationship between political ties and green innovation would be steeper when environmental regulations are more stringent*.

#### 3.2.3. Moderating Effect of Competitive Intensity on Business Ties

Competitive intensity is another external contextual factor that might affect the relationships between managerial ties and green innovation. It refers to the strength of competition in relation to the saturation level and growth potential of a market [67]. More intense competition has been associated with greater rivalry, more aggressive price wars, and competing product offerings [68].

The role of resources accrued through business ties in facilitating green innovation declines in a market with intensified competition. First, competitive intensity curtails the value and amplifies the risk of green innovation. When the number of players in the market and the aggressiveness of their behaviors increase, firms face the challenges of rapid product substitution, obsolescence, and upgrading [69]. Under such pressure, firms must identify market opportunities and turn them into real benefits in a timely manner [70]. However, as compared to other competing activities such as advertising expenditures and promotions, the rewards from green innovation are more uncertain and may take longer to materialize [7,40]. Therefore, amid a higher level of competitive intensity, firms tend to be more cautious when using their business ties to implement green innovations and prefer to take advantage of these resources to respond to the market rapidly.

Second, competitive intensity also accentuates the inefficiencies of business ties in conducting green innovation. Close business ties have the potential to preclude a firm from exploring new opportunities outside its current field [43]. This problem of inertia could be exacerbated by intense competition in the market that decreases the accuracy of forecasting and reduces the success rate of radical actions, and thus dissipating the firms’ willingness and capabilities to proactively make changes [68]. The firms are further encouraged to stay in their comfort zone rather than venture into unfamiliar areas such as green innovation that bear more risks. Therefore, we propose:

**Hypothesis 4a** **(H4a).** *The positive relationship between business ties and green innovation would be weaker when competitive intensity is higher*.

#### 3.2.4. Moderating Effect of Competitive Intensity on Political Ties

We also expect that competitive intensity could moderate the relationship between political ties and green innovation. Competition has aggravated the scarcity of resources that are available in the market [71], making the value of political ties in acquiring resources more salient under such arduous conditions. Exposed to higher levels of competition, firms with certain political ties may adopt more green innovations to address the authorities’ environmental concerns to guarantee the inflow of resources and support from the government. In addition, firms are more able to share the risks of green innovations with the government when strong bonds have been built between them, which has been especially critical in a hostile market [72].

However, competitive intensity could also magnify the negative effects of excessive political ties on green innovation. Heavy reliance on government-controlled resources and the shelter provided by the government cannot directly improve, and may even hamper, a firm’s innovative capabilities. It may also vitiate a firm’s motivation to engage in innovation, especially green innovation that requires greater commitment and is not bound to yield successful results [29,46]. These problems worsen under intensified competition. Second, fierce competition can distract the firm’s attention from long-term considerations [34]. When faced with external threats, firms with extensive political ties have been more inclined to exploit their government connections to avoid certain social responsibilities and instead embrace a set of utilitarian activities since eco-friendly processes or products may be of little value to gain advantages over their competitors in the short-term [52].

**Hypothesis 4b** **(H4b).** *The inverted U-shaped relationship between political ties and green innovation would be steeper when competitive intensity is higher*.

## 4. Methods

### 4.1. Data Collection

In order to test the proposed framework, we collected survey data from manufacturing firms in China. A key informant was identified for each firm, being someone who was a middle or senior manager working in the current firm for more than two years and was knowledgeable about the answers to our questionnaire. We distributed the questionnaires through site visit and e-mail in Xi’an, Tianjin, Beijing, and Yangtze River Delta region of China. A detailed explanation of the study was provided on the cover page. We delivered 500 questionnaires and received 276 replies after three reminders, of which, 218 were complete and satisfactory, accounting for a 43.6% response rate. The distribution of firms in our sample among industry sectors is provided in Appendix A (Table A1). We checked the non-response bias by comparing the responses of early and late waves of returned questionnaires in terms of attributes such as industry, firm size, and firm age. The t-tests did not yield any significant difference. Therefore, non-response bias is not a significant concern in our study.

To minimize the common method bias, we maintained full anonymity throughout the survey and gave the respondents assurance that there were no right or wrong answers. We also found that the single-factor structure fitted the data poorly in confirmatory factor analysis, which suggested that the common method bias did not appear to significantly influence the findings.

### 4.2. Measures

Well-established scales modified to our settings were used to operationalize constructs in the conceptual model. We employed the translation and back-translation technique to ensure the conceptual equivalence of our questionnaire. A few items that were considered ambiguous or inaccurate were revised after semi-structured and in-depth interviews with 10 senior managers for instrument validity. Before the final survey, a pre-test was conducted with 20 respondents in this field, on the basis of which we finalized our questionnaire. A seven-point Likert scale (1 = strongly disagree and 7 = strongly agree) was employed for all the measurement items except two control variables, i.e., firm age and firm size. Details of the multi-item constructs are provided in Appendix B (Table A2).

Following Rui and Lu [1] and Chen et al. [41], green innovation (GI) was assessed by seven items from three perspectives: overall management innovation, product innovation and process innovation. The two types of managerial ties were operationalized based on Guo et al. [18] and Peng and Luo [44]. The measure of business ties (BTs) comprised five items to capture the extent to which managers have developed good relationships with various market players, and the measure of political ties (PTs) comprised four items to capture the extent to which managers have developed good social relationships with government officials. We developed the measure of environmental regulations (ERs) based on Bhatia and Jakhar [37] and Peng et al. [73]. Five items were adopted to describe the regulations regarding environmental protection that have been implemented by government departments. Competitive intensity (CI) was assessed by four items modified from Jaworski and Kohli [68] and Chen and Liu [74], reflecting the strength of competition in the market.

We also controlled for several variables that could possibly affect corporate green innovation. Firm age (FA) was measured by the number of years since the firm’s establishment. For firm size (FS), the respondents were asked to indicate the number of full-time employees in their firms, based on six options: (1) less than 100; (2) 100–300; (3) 301–1000; (4) 1001–2000; (5) 2001–5000; (6) more than 5000. A two-item scale was employed to measure the extent to which a firm uses an innovation strategy (IS).

### 4.3. Measure Validation

Table 1 reports validity assessments of our questionnaire items. As it shows, Cronbach’s alpha for each multi-item construct is over 0.7, showing high internal consistency. Composite reliability (CR) of each construct measure exceeds the 0.8 threshold, and all average variance extracted (AVE) are greater than 0.6.

In terms of confirmatory factor analysis, a six-factor model was estimated for all the multi-item scales. The results indicated an acceptable fit with the data (CFI = 0.98; GFI = 0.88; RMSEA = 0.05). The statistically significant item loadings provided additional evidence for the presence of convergent validity.

Discriminant validity of the measures was assessed following Fornell and Larcker [75]. The squared correlation between each pair of constructs was less than the AVE for each individual construct, which suggested adequate discriminant validity.

### 4.4. Analysis and Results

The correlation matrix is presented in Table 2.

Since our model contains both the main effects of managerial ties and the interaction effects between managerial ties and contextual factors, we ran moderated regression models to test the hypotheses. To alleviate the potential threat of multicollinearity and facilitate interpretation, the scales used to construct the interaction terms were mean-centered before being multiplied.

A stepwise hierarchical approach was employed with three steps. In Step 1, we regressed green innovation on the controls to obtain Model 1. Step 2 added the predictors, i.e., business ties, political ties and political ties square, deriving Model 2. In step 3, the interaction terms between managerial ties and contextual factors were further included. In addition, following Sheng et al. [29], we added the interaction terms involving environmental regulations and competitive intensity separately, as shown in Model 3 and Model 4. This is because if all the interaction terms we hypothesized enter the model together, the potential for high correlations between interaction terms associated the same variable may overinflate the standard error of the regression coefficient estimates and render them insignificant. The variance inflated factor scores (from 1.05 to 2.64) suggested the absence of multicollinearity. The standardized regression results are summarized in Table 3.

With H1, we consider the effect of business ties on green innovation. Model 2 in Table 3 shows that BTs have a positive and significant effect on green innovation (β = 0.286, *p* < 0.001), thus supporting H1. H2 deals with the effect of political ties. As Model 2 shows, both the coefficients of PTs (β = −0.242, *p* < 0.01) and PTs^2^ (β = −0.185, *p* < 0.05) are negative and significant, suggesting that there is an inverted U-shaped relationship between political ties and green innovation, in support of H2.

**Figure 2:**
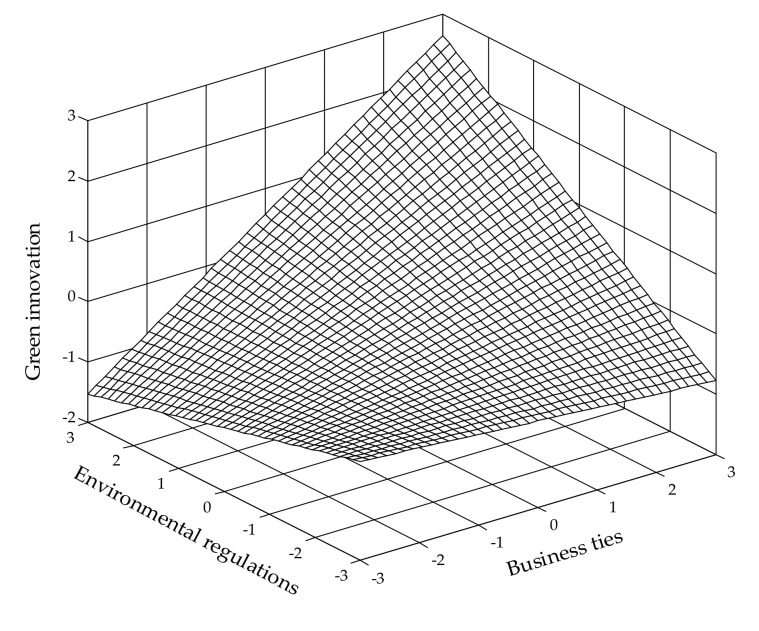
Moderating effect of environmental regulations on the relationship between business ties and green innovation.

In H3a, we expect a positive moderating role of environmental regulations on the effect of business ties. As shown in Model 3, the coefficient of interaction term between BTs and ERs is positive and significant (β = 0.128, *p* < 0.05), providing support to H3a. To clarify this moderating effect, we use the parameter estimates to depict it in Figure 2, which demonstrates a stronger positive effect of business ties on green innovation at high levels of environmental regulations than at low levels.

**Figure 3:**
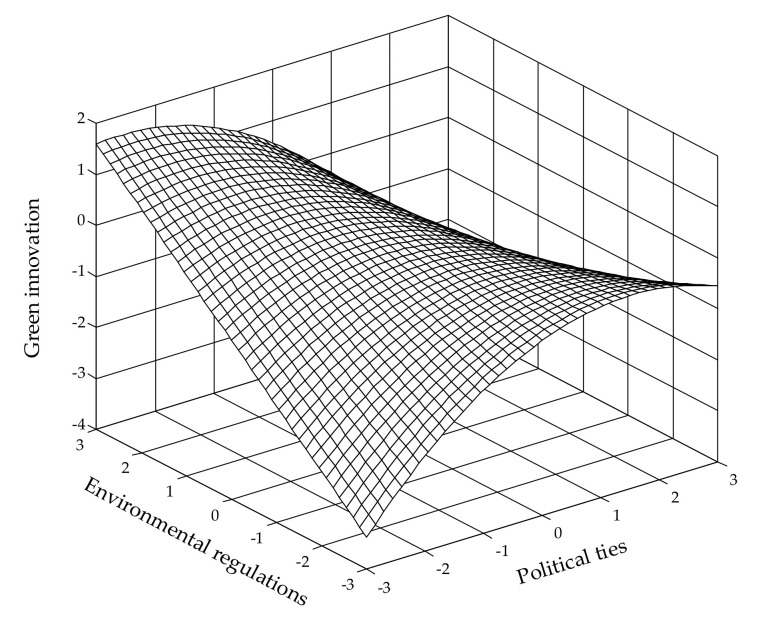
Moderating effect of environmental regulations on the relationship between political ties and green innovation.

H3b predicts the moderating role of environmental regulations on the effect of political ties. Model 3 shows that both the first-order interaction (β = −0.213, *p* < 0.01) and second-order interaction (β = −0.353, *p* < 0.001) between PTs and ERs are negatively related to green innovation, which indicates that environmental regulations strengthen the curvilinear effect of political ties on green innovation. Figure 3, which is based on these estimated coefficients, reveals that as environmental regulations get tougher, the inverted U-shaped relationship between political ties and green innovation becomes more pronounced. These results fully support H3b.

**Figure 4:**
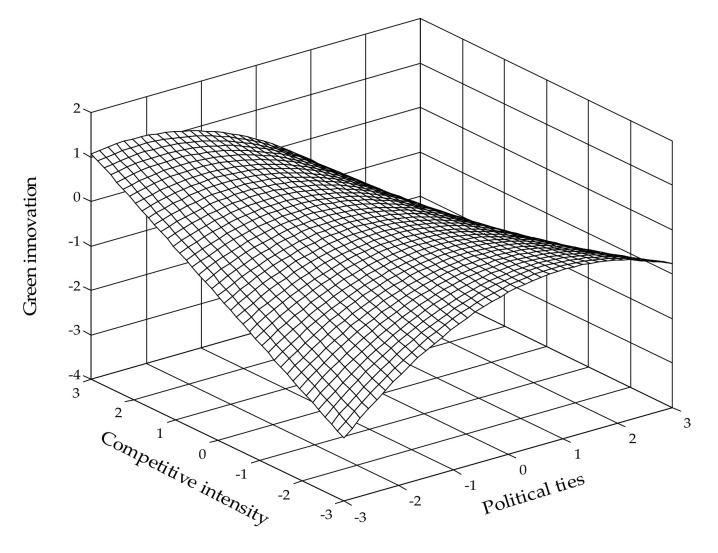
Moderating effect of competitive intensity on the relationship between political ties and green innovation.

In H4a, we argue that competitive intensity negatively moderates the relationship between business ties and green innovation; however, the interaction term between BTs and CI is not significant in relation to green innovation (β = 0.092, *p* = 0.178) in Model 4, providing no support to H4a. Finally, similar to H3b, H4b predicts that competitive intensity sharpens the inverted U-shaped relationship between political ties and green innovation. As shown in Model 4, both the coefficients of first-order interaction (β = −0.164, *p* < 0.05) and second-order interaction (β = −0.264, *p* < 0.01) between PTs and CI are negative and significant. Furthermore, Figure 4 describing the relationships among political ties, competitive intensity and green innovation illustrates that there is a steeper curvilinear relationship between political ties and green innovation when competitive intensity is high rather than low, lending support to H4b.

## 5. Discussion

### 5.1. Antecedents of Green Innovation

Our study brings social network perspective into the sustainability context and provides important insights for research on the antecedents of green innovation. The discussion about the role of social interactions on an interorganizational level in green development has been insufficient in the literature [2]. By linking green innovation to a firm’s social connections and identifying managerial ties as an important influencing factor of green innovation, we provide theoretical and empirical support for the concept that network-based factors have acted as important enabling mechanisms in the strategic choice of green initiatives [6] and add to the research that emphasized the involvement of external players in the ecosystem and the integrative capability for combining internal and external sources to achieve green innovations [20,25,76]. Particularly, open innovation strategies were hypothesized to provide positive impacts on eco-innovation, yet were found insignificant empirically [6]. Our findings may help explain this paradoxical result by showing that the cooperative innovation network must be properly designed and managed for open innovation strategies to make an impact.

### 5.2. Effects of Managerial Ties

The findings of our research enrich social network theory by indicating the relevance of managerial ties in a green management context. Prior research on managerial ties has mainly focused on how they affect a firm’s general innovation and performance [27,29] while the knowledge of their implications for environmental behaviors has remained limited. The significant roles that managerial ties have played in green innovation demonstrated by our research lend support to the value of social connections in tackling environmental concerns.

In addition, our results indicate how network composition could be used to predict green innovation. We distinguish between ties with various business organizations and ties with government entities, and find that they have heterogeneous effects on green innovation, reinforcing the assertion that these two types of ties capture distinct facets of social interactions and differ in content and ramifications [45,46,48]. Specifically, business ties provide firms with market resources, cooperation opportunities, risk-sharing mechanisms, and commercial reputation, and could positively enable it to adopt green innovation.

On the other hand, a small (but growing) body of research has investigated the role of political ties in the field of corporate environmental behaviors, yet with inconclusive results [12,34,51,52,54,65]. We reason and empirically confirm that cultivating political capital has been initially conducive to green innovation by ensuring the inflow of regulatory resources and exerting constraints on firms; however, overly close ties with the government and its affiliates distort firms’ resource allocation, weaken their incentives and abilities to implement green innovation, and even cover up their unethical profit-chasing behaviors at the expense of the environment. These effects have eventually led to a nonlinear inverted U-shaped relationship between political ties and green innovation. By taking a closer look at the pros and cons associated with political ties for green innovation, our results reconcile the divergent views in previous literature regarding the role of political connections.

### 5.3. Effects of Contextual Factors

First, our findings about the moderating effects of contextual factors advance the knowledge about the relationship between social networks and green innovation by revealing how it could vary under different situations. The positive effect of business ties and the inverted U-shaped effect of political ties on green innovation are both strengthened by environmental regulations. That is to say, business ties and moderate levels of political ties have better enabled firms to act in environmentally responsible ways if there are strong relevant regulations in place. However, coercive regulations have also worsened the defects associated with strong political ties and produced opportunities for abusing such ties, guiding the firms with excessive political ties to respond to the regulations in a counterproductive way. Therefore, the downsides of excessive political ties for green innovation are also more profound in the face of stricter environmental rules. These findings confirm the argument that it is important to associate innovation networks with the attribution of regulations [6].

Similarly, we find competitive intensity strengthens the positive effect of moderate levels of political ties, yet aggravates the negative effect of excessive political ties on green innovation as well. On the other hand, we hypothesized the value of business ties in inducing green innovation to be impaired in contexts with greater competitive pressure; however, this hypothesis did not receive empirical support. One possible reason is that business ties have enabled firms to take advantage of green innovation to fend off the competition. Currently, with increasing public consciousness of the environment and public health, as well as the decreasing effectiveness of conventional measures for competition, the benefits arising from green innovation, such as enhanced product advantages, boosted reputation, and expanded market share, have been increasingly valuable to earn competitive advantages and may even change the competitive rules [3,60]. Therefore, the firms facing fierce competition could make more use of their business ties to actively search for those otherwise neglected opportunities. In addition, ample resource repositories and reliable support provided by business ties could serve as a buffer against the risk in green innovation induced by market competition [18]. These reinforcing effects of competitive intensity on the relationship between business ties and green innovation could cancel out its dampening effects, leading to an insignificant moderating effect. To some extent, this result challenges the traditional view that green innovation is more of an action that aims to address the environmental sustainability rather than market competition [16,74] and corroborates that a changeable competitive market could create opportunities for sustainable enterprise practices [9].

Our findings concerning the moderating effects of contextual factors also lend some support to the Institution Difference Hypothesis (IDH), suggesting that the effects of social ties on strategic opinions and decisions regarding corporate environmental responsibilities could vary according to the level of economic and institutional development [77].

Second, previous work has largely focused on the direct effects of institutional pressures and market forces on environmental innovations, but debated on their efficacy, nonetheless. Regulations have been theoretically credited as effective instruments to promote eco-innovative propensities; however, the existing empirical evidence provided mixed results on the association between policy stringency and eco-innovation [16,36,62,78]. Similarly, some researchers have argued that green practices would be increasingly pursued in a more competitive market as its value in bringing distinctive advantages becomes more prominent while others have suggested that firms’ impetus to become greener would be weakened when they are faced with fierce competitions due to increased production costs and price of the goods [64,79]. Our findings contribute to resolving these disputes by delving into the previously understudied moderating effects of environmental regulations and competitive intensity. It is evidenced that they play significant yet intricate roles in green innovation when combined with the characteristics of firms and their networks, in agreement with the claim that policy actions and market pressures influence the persistence in eco-innovative attitudes and demand being intertwined with other domains of the firms’ economic activities in stimulating environmental innovative actions [6].

## 6. Conclusions

Green innovation that includes strategies intended to mitigate the negative environmental impacts of the firm’s activities has attracted attention around the world. It has the potential to improve a firm’s financial, environmental, and social performance [80], but, at the same time, it carries with it the double externalities problem, which could make a firm reluctant to become involved in such initiatives [42]. Combining social network theory, contingency view and green innovation literature, we develop a holistic model to examine the role of managerial ties in influencing corporate green innovative behaviors as well as how it varies under different institutional and task contexts. By doing so, we gain a fine-grained understanding of the relationships existing among different types of managerial ties, different types of contextual factors, and green innovation, providing implications for environmental practices.

### 6.1. Managerial Implications

Our findings have some implications that could enlighten managers on decisions regarding the development of managerial ties and green practices. They should acquaint themselves with the influencing mechanisms of each type of managerial ties on green innovation and the contingent effects of certain contexts, so as to effectively construct their external relationships for better green development.

First, green innovation is somewhat different from standard innovations. Our results suggest that managers should mirror its uniqueness when developing and utilizing social connections for green innovation. The two types of managerial ties play different roles in green innovation. On the one hand, business ties are conducive to green transformation, and accordingly, firms that intend to be more environmentally responsible should cultivate a good pool of business contacts to obtain more market resources and opportunities for green innovation. On the other hand, the benefits and risks inherent in political ties necessitate prudence when deciding the extent to which these ties should be relied on. Although good relationships with governmental agencies is important to create favorable conditions for green innovation, managers should also remain alert to avoid the dangers of overdependence on political ties. Specifically, they need to keep an appropriate distance from the government authorities and monitor the emergence of negative outcomes that could indicate the utilization of political capital has surpassed the optimal level.

Second, managers should adapt their network building and utilization strategies to the traits of institutional and task contexts in which the firms operate. Specifically, to respond to the environmental regulations, firms should rely more on business ties and a moderate level of political ties, the effects of which on green innovation can both be strengthened. On the other hand, across markets with different levels of competitive intensity, the effectiveness of business ties remains. In addition, to weather the increasing competitive intensity, firms should gradually place more emphasis on developing moderate connections with government officials for the benefit of green innovation. However, increasing reliance on political ties should be accompanied by discretion as its downsides can become more prominent under both stringent regulations and intensified competition. In a nutshell, network policies may play an important role and should not be a one-size-fits-all strategy in pushing firms to become environmentally responsible.

### 6.2. Policy Recommendations

Policy actions are compelling to promote economic prosperity encompassing social justice and environmental quality. This study also offers some suggestions for policy-makers to gain success for all from both micro- and macro-economic perspectives. As a possible direction to address the problems of resource shortage and environmental degradation, the governmental authorities should properly manage their relationships with firms to facilitate the environmental innovations of these firms, and be careful to avoid overly intimate relationships that would have negative consequences. It is necessary to reduce the dependence of firms on non-market advantages, and pay more attention to firms that have strong political backgrounds in case they shirk their environmental responsibilities and engage in pollutant behaviors under the cover of political ties. In fact, collusion between the firm and the local bureaucracy has been blamed for the pollution event of Zijin Mining, a Chinese listed company with political backgrounds. On the other hand, some fine tuning policymaking should be leveraged to promote connections between firms and the rest of the business community and to encourage the development of sustainable innovation ecosystems.

Meanwhile, it is the duty of governments to create high-quality institutional and market conditions through enhanced institutional design and implementation, so that firms can adopt eco-innovative practices based on the use of external ties. These two types of external contexts could potentially induce changes in the green strategy selection of enterprise. Strengthening the normative and informative roles of various environmental policy tools, such as environmental taxes, green R&D subsidies, and clean development mechanisms (CDM), could direct firms’ attention to environmental issues and encourage them to capitalize on their managerial ties to incorporate green management. We also recommend that governments should improve the quality of firms’ task contexts so the market could remain competitive and vigorous, spuring the firms to make better use of their social connections to assume more environmental responsibilities and undertake more green innovations.

### 6.3. Limitations and Future Lines of Research

Despite its contributions, this study has several limitations that should be addressed in the future. First, the cross-sectional design used did not allow us to establish the causality between predictors and responses or to explore the dynamic evolution of green development. Longitudinal designs would be suggested for future research if feasible. Second, our study mainly focuses on the relational dimension of social ties; other dimensions such as structural and cognitive ones could also play a role in green innovation, and they deserve theoretical and practical exploration. Third, different types of regulatory instruments in green management exist. It would be interesting to distinguish between them rather than treat them as a whole when investigating their moderating effects. Fourth, there may be other contingent factors that could influence the effects of managerial ties on green innovation, providing valuable directions for future research.

## Figures and Tables

**Table 1:** Construct reliability and validity.

Constructs	Scale Items	Factor Loadings	Alpha	CR	AVE
Green innovation (GI)	GI1	0.914	0.960	0.967	0.809
	GI2	0.831			
	GI3	0.914			
	GI4	0.867			
	GI5	0.933			
	GI6	0.913			
	GI7	0.921			
Business ties (BTs)	BTs1	0.874	0.946	0.959	0.822
	BTs2	0.892			
	BTs3	0.941			
	BTs4	0.930			
	BTs5	0.895			
Political ties (PTs)	PTs1	0.939	0.953	0.966	0.875
	PTs2	0.955			
	PTs3	0.924			
	PTs4	0.924			
Environmental regulations (ERs)	ERs1	0.836	0.946	0.959	0.826
	ERs2	0.952			
	ERs3	0.929			
	ERs4	0.903			
	ERs5	0.919			
Competitive intensity (CI)	CI1	0.632	0.767	0.858	0.604
	CI2	0.860			
	CI3	0.834			
	CI4	0.763			
Innovation Strategy (IS)	IS1	0.940	0.863	0.938	0.884
	IS2	0.940			

**Table 2:** Correlations.

Variable	1	2	3	4	5	6	7	8
1. GI								
2. FA	−0.11							
3. FS	0.04	0.31 **						
4. IS	0.08	0.07	−0.01					
5. ERs	−0.20 **	0.12	−0.16 *	0.08				
6. CI	−0.18 **	0.03	−0.11	−0.01	0.33 **			
7. BTs	0.34 **	−0.12	−0.04	0.06	−0.26 **	−0.28 **		
8. PTs	−0.22 **	0.09	0.06	0.08	0.22 **	0.26 **	−0.19 **	
Mean	4.25	12.15	2.72	5.43	4.80	5.10	4.92	5.00
S.D.	1.50	6.24	1.17	0.96	1.53	1.01	1.41	1.47

Note: ** *p* < 0.01, * *p* < 0.05.

**Table 3:** Hierarchical regressions results.

Variables	Green Innovation			
	Model 1	Model 2	Model 3	Model 4
Control variables				
FA	−0.113 (0.017)	−0.078 (0.016)	−0.005 (0.016)	−0.083 (0.016)
FS	0.041 (0.091)	0.062 (0.087)	0.003 (0.084)	0.057 (0.086)
IS	0.100 (0.103)	0.086 (0.098)	0.117 ^+^ (0.095)	0.108 ^+^ (0.098)
ERs	−0.144 * (0.070)	−0.059 (0.068)	0.188 * (0.091)	−0.054 (0.068)
CI	−0.121 ^+^ (0.104)	−0.004 (0.103)	−0.020 (0.102)	0.117 (0.133)
Direct effect				
BTs		0.286 *** (0.071)	0.314 *** (0.069)	0.291 *** (0.071)
PTs		−0.242 ** (0.076)	−0.170 * (0.074)	−0.188 * (0.077)
PTs^2^		−0.185 * (0.039)	−0.155 * (0.040)	−0.146 * (0.041)
Interactions				
BTs × ERs			0.128 * (0.040)	
PTs × ERs			−0.213 ** (0.042)	
PTs × ERs			−0.353 *** (0.025)	
BTs × CI				0.092 (0.063)
PTs × CI				−0.164 * (0.069)
PTs^2^ × CI				−0.264 ** (0.037)
Adj R^2^	0.050	0.160	0.230	0.183
F	3.275 **	6.184 ***	6.881 ***	5.421 ***

Note: *** *p* < 0.001, ** *p* < 0.01, * *p* < 0.05, ^+^ *p* < 0.1.

## Data Availability

Not applicable.

## Appendix A

**Table A1:** Distribution of sample firms among industry sectors.

Industry Sectors	Frequency	Percent
Automotive	33	15.1%
Electronics	43	19.7%
Electrical	32	14.7%
Mechanical	36	16.5%
Textile	23	10.6%
Pharmaceutical	25	11.5%
Chemicals	26	11.9%

## Appendix B

**Table A2:** Measurement items.

Constructs	Measurement Items
Green innovation (GI)	GI1: Our firm inclines to adopt new environmental management systems or methods.
	GI2: Our firm chooses the materials of the product that produce the least amount of pollution.
	GI3: Our firm uses the least amount of material to comprise the product.
	GI4: Our firm circumspectly deliberates whether the product is easy to recycle, reuse, or decompose.
	GI5: The manufacturing processes of our firm effectively reduce the emission of hazardous substances or waste.
	GI6: The manufacturing processes of our firm effectively reduce the consumption of energy or resources.
	GI7: The manufacturing processes of our firm effectively recycle the waste and emission that can be treated and reused.
	Managers of our firm have developed good relationships with managers of
Business ties (BTs)	BTs1: Suppliers.
	BTs2: Customers.
	BTs3: Competitors.
	BTs4: Marketing-based collaborating firms.
	BTs5: Technological collaborating firms.
Political ties (PTs)	PTs1: Managers of our firm have maintained close relationships with government officials at various levels.
	PTs2: Managers of our firm have established close relationships with officials in regulatory and supporting organizations such as state banks, commercial and industrial administration bureaus.
	PTs3: Our firm’s relationships with local government officials are satisfactory.
	PTs4: Our firm has allocated a great deal of resources to build relationships with government officials.
Environmental regulations (ERs)	ER1: Government regulations concerning the environment, such as waste emission and cleaner production, are stringent.
	ER2: There is environmental oversight from the government.
	ER3: Environmental policies are systematic and specific.
	ER4: The intensity of environmental regulations is increasing.
	ER5: Existing punishments in the environmental regulations are severe.
Competitive intensity (CI)	CI1: Price competition is a hallmark of our industry.
	CI2: Any action that a firm takes, others can make a response to swiftly.
	CI3: One hears of a new competitive move almost every day.
	CI4: There are many promotion wars in the market.
Innovation Strategy (IS)	IS1: Our firm places emphasis on R&D through allocation of substantial financial resources.
	IS2: Our firm places emphasis on increasing the rate of innovation.
